# Adherence of those at low risk of disease to public health measures during the COVID-19 pandemic: A qualitative study

**DOI:** 10.1371/journal.pone.0276746

**Published:** 2022-10-25

**Authors:** Gemma Postill, Cindy L. Adams, Claire Zanin, Michael Halpin, Caroline Ritter

**Affiliations:** 1 Temerty Faculty of Medicine, University of Toronto, Toronto, ON, Canada; 2 Veterinary Clinical and Diagnostic Sciences, University of Calgary, Calgary, AB, Canada; 3 Faculty of Science, University of Guelph, East Guelph, ON, Canada; 4 Department of Sociology and Social Anthropology, Dalhousie University, Halifax, NS, Canada; 5 Department of Health Management, University of Prince Edward Island, Charlottetown, PE, Canada; University of Exeter, UNITED KINGDOM

## Abstract

Public health measures (PHMs) proactively and reactively reduce the spread of disease. While these measures target individual behaviour, they require broad adherence to be effective. Consequently, the World Health Organization issued a special appeal to young adults, a known non-adherent population, for increased adherence with COVID-19 guidelines. However, little is known about why these low-risk individuals do or do not adhere to PHMs. This study investigates why young adults in a low-risk setting adhered to PHMs implemented during the COVID-19 pandemic. A qualitative research approach was chosen to gain an in-depth understanding of participants’ thoughts and experiences related to PHM adherence. Semi-structured interviews were conducted in April-May 2021 with 30 young adults living in Prince Edward Island (PEI), the province with the lowest COVID-19 case rate in Canada at that time. Thematic analysis was used to create a codebook based on the Theoretical Domains Framework, which was then inductively modified. The analysis identified eight themes that explained the adherence of young adults: (1) clear, purpose-driven adherence rationale, (2) developing trust in the local leadership, (3) adapting to novel measures, (4) manageable disruption, (5) adhering to reduce anxiety, (6) collective duty towards one’s community, (7) moral culpability and (8) using caution rather than compliance. Together, these themes demonstrate that young adults adhered to PHMs because of their sense of connection to their community, public health leadership, and concerns over stigma. We further argue that clear guidelines and communication from public health officials during both periods of high and low COVID-19 cases facilitate adherence. These findings are important for mitigating future public health emergencies as they explain why young adults, an important segment of the population whose adherence is critical to the success of PHMs, follow PHMs. Further, these findings can inform public health officials and other stakeholders aiming to develop successful adherence strategies.

## Introduction

Public health measures (PHMs) are actions taken by governments, organizations, or individuals to reduce the spread and burden of disease. Numerous PHMs were used to mitigate the spread of Sars-CoV2 (i.e., COVID-19), a novel respiratory virus that spread globally, being declared a pandemic by the World Health Organization (WHO) on March 11, 2020 [1]. The PHMs used for the COVID-19 pandemic ranged from symptom checking to mandatory isolation orders [2] and differed between countries and local jurisdictions.

While PHMs target the behaviour of individuals, their success is dependent on the adherence of everyone [3–5]. Research suggests several socio-psychological factors that motivate or deter adherence to PHMs. For example, perception of risk [6], social integration [7], empathy [8], and social responsibility [9] are factors that facilitate adherence. Previous research also identified non-compliant populations including males [10, 11], young adults [9, 11, 12], individualistic [13] and/or impulsive [14] individuals, and those with antisocial personality traits [15].

Despite this extensive literature, less is known about why those at low-risk adhere and whether the same motivators and deterrents of adherence are applicable to all PHMs. This is because previous literature focused on (1) adherence in relation to a single PHM and (2) quantitatively analyzing factors that motivate or deter adherence rather than the process of adherence. While these studies provide valuable information, qualitative research is needed to capture the humanism of adherence and enable an in-depth understanding of why individuals adhere. Likewise, much of the previous adherence research employed national or even multi-national participant recruitment, which provides less information as to how adherence unfolds in specific contexts, which is important as the level of risk and PHMs used varies between regions. Prince Edward Island (PEI) is a Canadian province with the lowest case rate of COVID-19 in Canada in early 2021 [16]; and consequently, provides a unique low-risk environment to study compliance situated in a context with lower risk (S1 Appendix). We are unaware of any research describing adherence behavior of PEI residents with the exception of adherence to a school nutrition program [17].

Young adults have been identified as a non-adherent population [7, 18], but there is considerable debate about why young adults are non-adherent. Some argue young adults’ large social networks and active social lives mean their adherence requires more substantial lifestyle changes than for older individuals [19, 20], while others have proposed that young adults’ low self-control, impulsivity, and certain personality traits facilitate non-adherence [12, 21, 22]. Understanding the reasons for (non-) adherence in young adults is critical because this group as a whole makes up a significant portion of the population, has large social networks, and is more risk tolerant than older adults [10], making them more likely to transmit the SarsCoV-2 virus and other infectious diseases. Recognizing young adults’ potential to substantially contribute to virus transmission rates, the WHO issued a special appeal for increased adherence with COVID-19 guidelines among young people [23]. The Centers for Disease Control and Prevention also highlighted the need to motivate adherence to PHMs among this population given they were more likely to transmit the virus [24].

Consequently, there is a need for research approaches that employ qualitative inquiry to understand the adherence of young adults to PHMs. The objectives of this study were thus, to (1) understand the socio-psychological motivators and deterrents of adherence for young adults in the COVID-19 pandemic and (2) understand differences and similarities of the factors affecting adherence across the various PHMs.

## Materials and methods

### Research approach

A qualitative research approach was chosen because of its strength in generating knowledge grounded in human experience [25]. Specifically, we collected qualitative data through semi-structured interviews between the researcher (G.P.) and participants. The use of semi-structured interviews enabled the interviewer to guide the discussion while also permitting the discussion of other topics important to the research question in greater detail as they arose [26].

### Participant recruitment & description

The study population consisted of English-speaking individuals in their 20s currently living in Prince Edward Island (PEI; a Canadian province). In accordance with the research objectives, we sampled young adults to understand adherence to different PHMs. Young adults were defined as individuals 20–29 years old, as some of the PHMs, namely provincial COVID-19 testing recommendations, specifically targeted those aged 20–29 years old [27]. Thus, this group was chosen as individuals experienced a similar risk and were exposed to the same PHMs. At the time of the first interview (April 13th, 2021), PEI reported 163 total COVID-19 cases among a population of approximately 159,000 [16]. Most cases were travel-related, there was little community spread, and no deaths directly attributable to the Sars-CoV2 virus. As such, this study design enabled us to understand adherence in an extremely low-risk environment.

During recruitment we labelled the study as an analysis of “pandemic experiences of young adults” to avoid attracting only individuals who adhered to PHMs and influencing participant responses during the interviews. Participants were recruited in three ways. First, we posted a recruitment letter in a public *Facebook* group called *Ask PEI* that had approximately 14,500 members at the time; these members are generally either living in or have lived in PEI. Second, we used a radio advertisement through *Island News*, the *CBC Radio* Station in PEI. From these methods, 25 participants were recruited for the study. However, as this convenience sample was comprised of mostly women, five men were additionally recruited through network sampling [28]. Specifically, the previously interviewed participants were approached and asked if they could refer a male friend, family member, or acquaintance to the study. The choice of 30 participants was made during the interviewing process as data saturation occurred at that point. Data saturation occurs when additional interviews cease producing new themes [29].

### Development of the semi-structured interview guide

Our semi-structured interview guide (S2 Appendix) consisted of key questions that guided the discussion [26]. Creating the semi-structured interview guide was an iterative process that involved a review of relevant literature, assessing the question guides of similar qualitative studies, and discussing the guide amongst the research team. Then, the interview guide was pilot-tested with 3 individuals who were not included in the analysis; the feedback generated from this testing resulted in minor changes to the interview guide.

The final interview guide consisted of the following sections: (1) perceptions, attitudes, and beliefs in relation to adherence with COVID-19 PHMs, (2) rationale for (non-) adherence to each individual request (i.e., COVID-19 testing and social isolation) within the circuit breakers (periods of temporary provincial shutdown, as explained in S1 Appendix), as well as retrospective accounts on how adherence changed from circuit breaker to circuit breaker, and (3) attitude towards getting vaccinated against COVID-19.

The specific PHMs included in the interview guide were mask wearing, social distancing, 14-day post-travel-quarantine, circuit breakers, and vaccination, as they were the most prominent regulations used in PEI during the COVID-19 pandemic (S1 Appendix). Throughout the interviews, the question guide was modified slightly in response to the participants’ impressions and thoughts (e.g., early participants described compliance on a scale of 1 to 10, and thus, the interviewer G.P., added a questions to specifically ask subsequent participants’ to rate their compliance on a scale) [29].

### Data collection: Semi-structured interview

The interviews took place during April-May 2021, between PEI’s fourth and fifth pandemic waves (S2 Appendix). The majority of interviews occurred over the teleconferencing software *Zoom* [30], with the remaining on the telephone (N = 3) by request of the participant. Of those interviews over *Zoom*, most participants (N = 24) completed the interview with the camera on. All interviews were led by the same researcher (G.P.), whose video remained on regardless of the participant choice to ensure consistency across interviews. We used online videoconferencing for interviews given that at the time, public health guidelines discouraged in-person meetings, and previous research had shown the use of *Zoom* for individual interviews had similar quality to in-person interviews and was preferred by participants to other forms of interviews, including in-person interviews [31, 32].

Before the interview, participants provided either recorded verbal or written consent (i.e., through a signed consented form) and then completed a short (<5min) demographic questionnaire (S2 Appendix). The questionnaire also included seven COVID-19 knowledge questions, which helped to contextualize the participants’ knowledge of COVID-19. Following the questionnaire, participants were led through the semi-structured interview guide. The interviews ranged in length from 47 to 88 minutes. We recorded short analytic notes (i.e., memos [30] after each interview and throughout our analysis of the transcripts to log researcher notes and perceptions related to the study objectives [33]. Audio-recordings of the interviews were transcribed verbatim either by G.P. or by a professional transcription service who signed a confidentiality agreement. G.P. completed accuracy checks of all the transcripts.

### Data analysis

Deductive and inductive thematic analysis was used to analyze the transcripts [34, 35]. Coding was done by G.P. and selected transcripts were reviewed and discussed by the larger research group in weekly research meetings, ensuring that all researchers immersed themselves with the data [34, 35]. Meeting minutes were recorded to establish an audit trail.

We deductively coded transcripts in relation to the Theoretical Domains Framework (TDF). The TDF is used to provide a systematic, structured approach for integrating a multiple of well-supported theories on health behaviour. It is an integrative framework consisting of 14 domains that synthesize 33 different psychological theories (e.g., Social Cognitive Theory and Modelling) and 128 constructs related to behaviour change [36, 37]. The use of the TDF framework for the analysis of health behaviours and adherence has been well-documented in previous qualitative studies such as pregnancy weight management [38], personal decisions for health checks [39], and clinicians’ genetic testing practices [40]. Systematic reviews have also shown the TDF to enables an analysis of the deterrents to adopting new health behaviours (e.g., responding to new healthcare guideines) [41].

Following the recommendation of Atkins et al. (2017), we analyzed the data deductively, using the TDF to categorize the initial codes found in the transcripts [39]. After we completed our deductive coding, we conducted an inductive analysis, to ensure that sentiments expressed by participants that did not fit neatly into the TDF were considered. Our combination of inductive and deductive analysis is consistent with prior TDF literature, which emphasizes that while the TDF framework is comprehensive, as it contains several behaviour change theories and constructs, it might not be exhaustive [42]. The inductive and deductive codes were considered together, and themes were generated from these codes through an iterative process by which codes with similar sentiment (as opposed to theory or content, the approach in the TDF) were grouped together and labelled according to the meaning conveyed by participants. The final codebook consisted of eight themes with 19 parent codes and 52 child codes across these themes (S3 Appendix).

In reporting extracts of raw data, pseudonyms are used. The participant’s gender (M for Man, and W for Woman; no other genders were reported) and age are stated along with the pseudonym to contextualize the statement: for example, John (M, 21). Member checking was done following the synthesis and reporting of the results to ensure the experiences of all participants were captured [43].

### Researcher description

This study takes a critical realist approach. Critical realism suggests that one reality exists, which can never be understood perfectly. Nonetheless, research and its theories can help to explain causal mechanisms underlying social mechanisms or phenomena [44]. As one’s lived experience influences their positionality, the authors also reflected on their positionality throughout the research. C.R. lived in PEI during the pandemic, C.A., C.Z., and M.H. experienced the pandemic living in other Canadian provinces, and G.P. lived in both PEI and outside of PEI during the pandemic.

**Ethics approval.** This study was approved by the ethics board of the University of Prince Edward Island (REB #6009061).

## Results

### Participant characteristics

Participants were young adults (aged 20–29 years) living in PEI who varied in key demographic factors (see Table 1). None of the participants had contracted COVID-19 (to their knowledge) at the time of the study. The participants demonstrated knowledge of COVID-19, including knowledge of common symptoms, risk factors and transmission pathways, based on the administered knowledge test, with a median and mean of six correct answers (range: 4–7 correct) out of the seven questions.

**Table 1:** Demographic information of the 30 young adults participating in the individual interviews.

Demographic		n	%
**Gender** (open text box)	Man	11	36.7
	Woman	19	63.3
**Born in Prince Edward Island**	Born in Prince Edward Island	18	60.0
	Not born in Prince Edward Island	12	40.0
**Highest level of education**	High school	7	23.3
	College Diploma/Undergraduate degree	16	53.3
	Masters or Post-graduate degree	6	20.0
	No response	1	3.4
**Current student status (University or College)**	Current student	10	33.3
	Non-student	20	66.7
**Employment status**	Not employed	5	16.7
	Part-Time	5	16.7
	Full-Time	20	66.6
**Type of dwelling**	Condominium/Apartment	11	36.7
	Detached House	15	50.0
	Duplex/Townhouse	4	13.3
**Marital status**	Married or common law	11	36.7
	Single or non-cohabitating relationship	19	63.3
**Cohabitating: Number of other people in household**	0	5	16.7
	1–2	19	63.3
	3–6	6	20.0
**Cohabitating with those >60 years**	No	25	83.3
	Yes	5	16.7
**Cohabitating with immunocompromised people**	No	25	83.3
	Yes	5	16.7
**Cohabitating: Number of children**	No	29	96.7
	Yes	1	3.3
**Age (mean, median, range)**	24.4, 24, 20–29		

### Rationale for adherence to the various public health measures

The participants described being generally adherent with the provincial PHMs studied, specifically mask wearing, social distancing, 14-day post-travel quarantine, circuit breaker requirements, and vaccination (S2 Appendix). When asked during the interview to rate their overall adherence (one meaning they did not follow any rule that required behaviour change and ten meaning followed every rule perfectly), the most common answer reported was a seven out of ten (median = 7.5; range of 6 to 10). Participants gave themselves this score given they were largely compliant, but also found themselves in situations where they chose to bend the rules (i.e., to meet with more people than recommended and/or to not socially distance around those one knew well).

**Fig 1:**
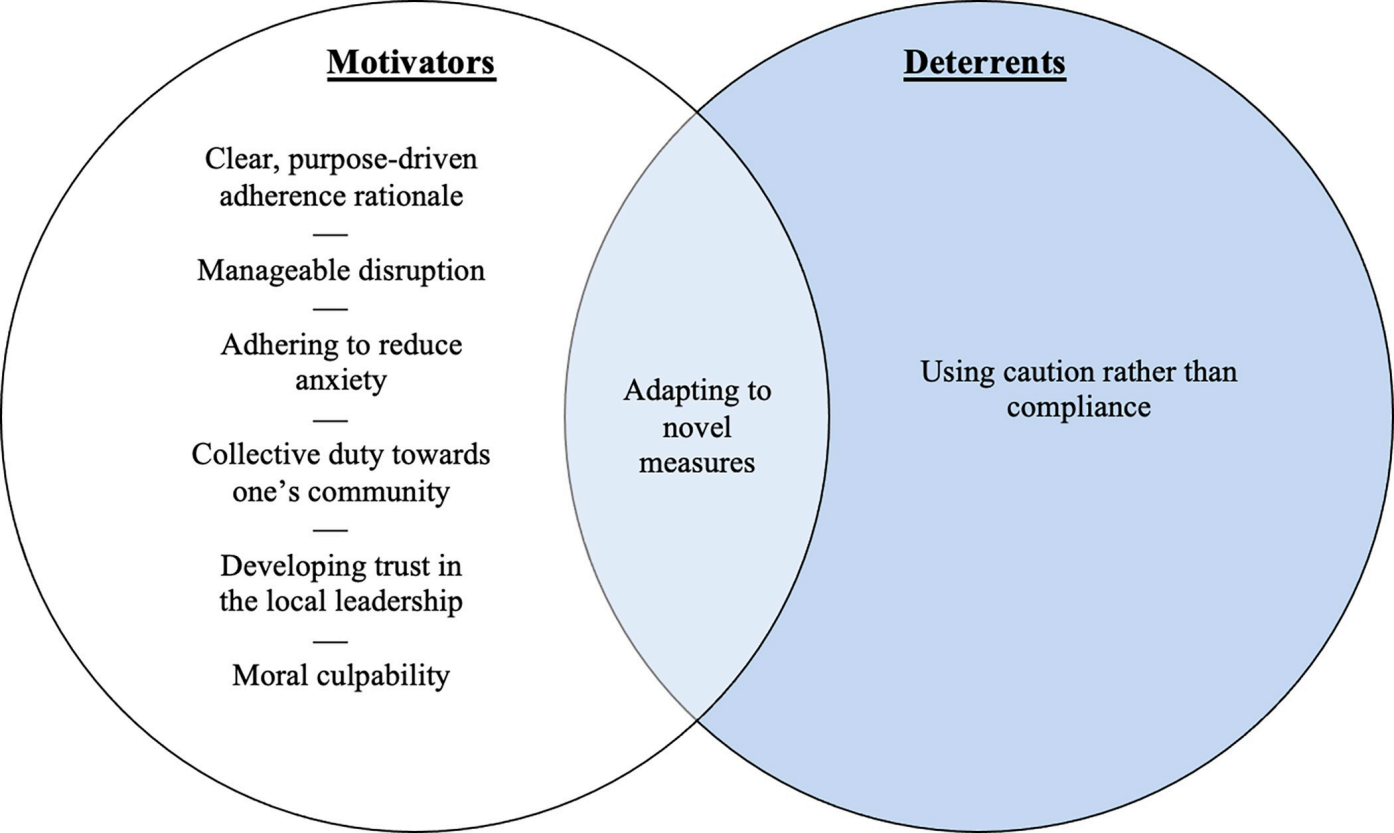
The common themes identified across participants and whether they were motivators or deterrents for adherence with COVID-19 public health measures.

Our analysis of why young adults living in a low-risk environment chose to adhere or not with precautionary PHMs revealed eight themes (Fig 1). These themes were reflective of the adherence behaviours of all participants and included: (1) clear, purpose-driven adherence rationale, (2) developing trust in the local leadership, (3) adapting to novel measures, (4) manageable disruption, (5) adhering to reduce anxiety, (6) collective duty towards one’s community, (7) moral culpability and (8) using caution rather than compliance. We broadly classified these themes as motivators or deterrents of adherence. However, the analysis also revealed nuances between the specific PHMs.

### 1 Clear, purpose-driven adherence rationale

The young adults interviewed adhered to a PHM when the purpose of the PHM was clearly explained by public health officials and thus, perceived as necessary and executable. Having a clear purpose-driven rationale helped to justify challenges of adherence. For example, Tariq (M, 27) described that he complied to the circuit breakers because, “the way they presented the information I felt was very clear, and I felt I had a good understanding of […] what I had to carry out to comply with the circuit breaker. So, I felt it was clear, and it was justified in that the consequences of it were clear.”

Descriptions young adults used to describe the specific purposes and effectiveness of PHMs that they complied with included “returning to normal”, “flattening the curve”, or achieving “COVID-Zero”. For participants, some of these goals were more pertinent to specific PHMs (i.e., mask-wearing was perceived to help with “flatting the curve” while circuit breakers helped with achieving “COVID-Zero” states); however, despite the different goals, young adults still complied to the different PHMs. For example, all participants connected their adherence for mask-wearing and vaccination to a desire to maintain in-person activities and social gatherings. Alfred (M, 21) justified his vaccination adherence by stating, *“*six months down the line it’ll be all worth it because we’ll be a little bit closer to normal or whatever normal is at that point.” In contrast, when explaining adherence to the 14-day quarantine for off-island travel and circuit breakers, young adults referenced the goal as reaching “COVID Zero” (e.g., young adults followed the circuit breaker rules in order to have no cases, so that they could see friends and family over the December holidays). Such, statements about “COVID Zero” were not directly brought up in regards to mask-wearing, which highlights that the young adults adhered to the different PHMs for different purposes. Taken together, young adults at low-risk described being more likely to follow an intervention when it is perceived as useful or purpose-driven (i.e., achieves a public health goal).

### 2 Developing trust in the local leadership

Young adults adhered with the PHMs because of the trust they developed in the local leaderships throughout the pandemic. As with other provinces and territories in Canada, the PHMs during the pandemic were communicated by both public health officials and government leaders. In PEI, the pandemic rules were largely communicated by Dr. Heather Morrison, the Chief Public Health Officer of PEI, in weekly briefings that were broadcasted live over Facebook. Most participants described focusing the information intake on Dr. Morrison’s public health briefings:

I definitely trusted them [public health officials]. I feel like it [the weekly briefings] turned into this ceremony where everyone checked in to see what they were saying about how many cases. You had this bond with each other. You had to get on Facebook and say you’re watching those–so maybe like just seeing them on the screen and that kind of transparency was probably a helpful thing. It was like a get to know these public health people that protect us all. Tyler (M, 22).

This quote highlights that consistent communication from public health officials facilitated the strengthening of community membership, which helped young adults to be more acutely aware of the changing local situation and fostered trust in the public health officials as they were seen to be transparent. Tyler (M, 22), as well as the others interviewed, went on to explain that they were more likely to adhere to new guidelines because “I just trusted what the public health people were saying, eventually. I was like, okay, if they want me to do it, then it must be helping. It must work…” Therefore, the trust in the local leadership, fostered through transparent and consistent communication about the status of COVID-19 cases and necessary PHMs, encouraged young adults to adhere to public health guidelines.

### 3 Adapting to novel measures

The newness of the pandemic rules was both a barrier and a motivator to adherence for young adults (Fig 1). Initially, young adults perceived adherence to new measures as more challenging, especially when adherence to a new measure required behaviour change. For example, young adults described challenges in adopting masks because of the change it had on their routine and social interactions. Javier (M, 23) described this saying, “Having to wear a mask throughout my 12-hour shift at work is also–well now it doesn’t bother me, it just feels like second nature–but at first it was a big, big, big change. […] It changed the way we interact with [clients/patients].” None of the young adults interviewed described the novelty as an absolute deterrent; rather they described novelty as an obstacle that they had to work around.

The difficulty in adhering to new measures were exacerbated when new guidelines were perceived as vague (e.g., not clearly communicated); such findings relate to theme 1 (e.g., clear, purpose-driven rationale). Yasmin (W, 29) described this, saying, “Sometimes I feel that the definition of a guideline is very vague […] that makes it very difficult.”

In contrast, young adults also described that novel PHMs encouraged adherence (Fig 1). For example, young adults were unaware of what circuit breakers were prior to their implementation in December 2020. Participants described the announcement of the circuit breaker as novel and fear-inducing, analogous to the beginning of the pandemic in Spring 2020. However, the interviewed young adults described notable differences in their adherence with the second circuit breaker (February 2021); specifically, they lacked the same level of motivation to completely follow isolation rules as the novelty had worn off (Fig 1). Theresa (F, 22), described this saying, “Fear has decreased in PEI […] [We] are just like complacent.” This quote highlights the interconnectedness of themes of novelty and anxiety; thus, as the initial fear and anxiety subsided, the novelty wore off and was replaced with COVID-19 fatigue as time progressed from the implementation of a PHM.

Therefore, in our data, the novelty of measures was connected to adherence and non-adherence; novelty was more likely to prompt adherence when the rules were explicit, the rationale was clear, the behaviour change was defensible, and there were mechanisms to remind those who forget or who have difficulty adhering with public health rules.

### 4 Manageable disruption

Interviewed young adults adhered to PHMs because they believed the change or disruption caused by adherence was manageable (Fig 1). For example, Theresa (W, 22) described, “all I have to do is wear a mask and I can still go to dinner, like I’m all good with that.” Perceiving the disruption as manageable often stemmed from the perception that adherence was the normative choice of action. This finding reinforces that of theme 3 (e.g., adapting to novel measures) as it demonstrates that priming young adults with examples of others adhering motivates adherence. For example, Alfred (M, 21) described how masks had become part of the Atlantic culture by the time PEI introduced mandatory indoor mask wearing policies in November 2020 by saying, “I remember going to Nova Scotia (a Canadian province) in the summer and at that point in the summer they had a mask mandate well before PEI did, so I kind of got used to it there. By the time it came into effect here in PEI I felt good about it… it was weird not to wear a mask.” Thus, seeing other neighbouring jurisdictions adopt similar policies, like mask wearing, led to the belief that adherence was not difficult. Participating young adults were also more likely to perceive self-efficacy and the disruption manageable, and then adhere with a specific PHM when adherence was seen as a normative action in PEI or in neighbouring jurisdictions.

### 5 Adhering to reduce anxiety

Participants adhered to PHMs to reduce their anxiety (Fig 1). Uncertainty was a main source of anxiety, as described by Amy (W, 24), who said “it was ‘the unknown’ that everyone was really scared about.” Recognizing the global connectedness and potential for spread in a pandemic, young adults recognized that a COVID-19 case could still arrive in PEI. Michael (M, 27) stated “there were so many unknowns that the gathering limit seemed completely like normal and necessary.” Young adults attributed the ability of guidelines to alleviate anxiety to the fact that in PEI, public health officials, as opposed to elected government officials, delivered the public health messaging; thus, early on in the pandemic, young adults believed that in light of the uncertainty early on in the pandemic that most PHMs were enacted purely to ensure public safety.

A subset of participants described strong feelings of anxiety around the pandemic and indicated that these feelings prompted them to use precaution beyond what was asked by public health officials. This was the case for Susan (W, 29) who recalled “I got one big three-week grocery order instead of like a one week at a time sort of a thing. We didn’t go into any stores, even though the stores were open. I just didn’t want to kind of risk it.” Thus, young adults’ anxiety and fear of the unknowns legitimized the rules, making adherence to the new PHMs seem necessary.

### 6 Collective duty towards one’s community

Young adults also adhered to PHMs because they had a sense of responsibility to the community (Fig 1). Bethany (W, 28) summarized this need for collectivity by saying, “I understand the regulations that need to be followed… I see that they work, and I’m very happy to be part of that collective group to help keep people safe.” Young adults also acknowledged that underlying instabilities in the PEI healthcare system made adherence and collective action necessary. Jeffrey (M, 24) described this saying, “On the best of days we’re a very strained healthcare system. […] Even just one of these self-contained large outbreaks could completely shatter the fragile system we have.” Similar to other participants, Jeffrey described how this knowledge prompted them to want to adhere with guidelines.

Participants were proud of their ability to uphold this commitment to community, as articulated by Susan (W, 29) who said, “I’d like to think that we were kind of the envy of Canada—how we handled things and kind of kept most of our kind of daily routines and freedoms that we would have had beforehand so.” This pride motivated participants to continue maintaining “zero COVID-19 cases”. For example, Linda (W, 26) described how they knew that as soon as there was community spread, there was a need to increase the public health restrictions. “When case counts started to go up, I knew they were going to do something because I mean the strategy is definitely getting to basically zero COVID cases on the Island at all times.” Therefore, young adults’ motivation to adhere was tied to their integration within their community and the sense of responsibility that such entails.

### 7 Moral culpability

Young adults also adhered with the guidelines, despite their being a low risk, because they felt that they would be culpable if the outcome of their action was spreading COVID-19 to others (Fig 1). Several participants described this culpability as serious enough to be labeled irresponsible for potentially hurting someone else. For example, Kelsey (W, 21) said that being caught spreading COVID-19 would be “like smoking in front of your kids, or texting and driving”. Young adults chose to adhere with the guidelines to avoid the guilt and culpability from being in those situations, as stated by Christina (W, 28), who said, “I know a lot of people have gone through it inevitably–passing things on to people who later die. I feel like I would feel guilty for the rest of my life. I don’t know what it is necessarily like.” Consequently, young adults in low-risk settings were motivated by moral culpability of non-adherence.

The moral culpability of spreading COVID-19 was reinforced by the formal (i.e., fines) and informal (i.e., social judgement) policing of non-adherence. For example, Josh (M, 27) described the social stigma around breaking the rules saying, “They name-dropped a guy. The first person to be charged with [breaking COVID-19 rules] […] I think just the fact that there were legal repercussions was telling.” Xavier (M, 22) described the informal policing during circuit breakers when he said, “You don’t want to be caught without your [COVID-19] test or your mask.” Likewise, Sally (W, 28), described the labeling associated with the informal policing when she said, “my parents have a word for people that don’t follow the rules, and they’re called cov-idiots.” Such statements demonstrate the stigma associated with non-adherent behaviour, which motivated participants be adherent to PHMs to avoid becoming labelled as deviant.

### 8 Using caution rather than compliance

Young adults sometimes chose not to adhere to PHMs, favoring the use of caution rather than direct compliance to the rules in order to maintain their existing daily habitus and social relationships (Fig 1). For example, Shelley (W, 29), described choosing not to adhere in order to gather with friends and family, “You could only be around 10 people outside of your household and it was supposed to be consistent. It definitely wasn’t consistent [for me] and I knew that it wasn’t consistent for the other person.” Likewise, George (M, 21) described a similar sentiment saying, “I probably would still be doing stuff–spirit of the law instead of rule of the law.” By this Shelley and George referenced an internal negotiation undertaken by most young adults in which they considered the goals of PHMs, and acted in line with such goals, but did not adhere to the specifics of the PHM, which we refer to as employing caution instead of compliance.

Young adults described complying to PHMs when with strangers and using caution instead of compliance when among friends, especially those that are transparent about their other social activities. For example, Tariq (M, 27) described this saying, “obviously we don’t go around like high fiving strangers, but at the same time you still high five your friends you know?” In addition to social gathering limits, this was the case for mask wearing; wearing a mask was normative around strangers but seemed unnecessary with friends.

Regardless of whether they adhered to the gathering limits, all young adults described a perceived low-risk to gathering with close friends because of the low number of COVID-19 cases, the other guidelines in place, and the reduced likelihood of adverse events for young adults. For example, Michael (M, 27) said, “It was definitely—you know it was like a calculation of risk, right? […] With the current rules that are in place and the fact that I didn’t know anybody that had COVID I felt like that the risk of me getting COVID-19 was extremely low.” Thus, the perceptions of low risk and invulnerability led to young adulting being less likely to follow the rules over time, and instead just employ caution, especially when with friends.

## Discussion and conclusion

This paper explains why young adults in low-risk environment did and did not adhere to PHMs. Taken together, the identified themes we demonstrate specific rationales for adherence behaviour that policy makers can target. As the success of PHMs depends on the adherence of those at low risk, the findings presented here are useful to the coordination of PHMs in future public health emergencies in order to effectively preventing the spread of disease. Further, we demonstrate connections between themes. Specifically, the rationales for adherence compound with one another; for instance, novelty, community pride, and clarity can all buttress one another. Such interrelations highlight that when public health official experience difficulty in motivating adherence (e.g., due to “COVID-fatigue”), they can draw on the other strategies presented here, such as developing trust and community.

The novelty of the COVID-19 pandemic is an important motivator of adherence. The uncertainty surrounding a new public health threat creates immense anxiety among young adults, even those in extremely low-risk environments. These findings are consistent with quantitative survey data conducted at the onset of the pandemic [6]. Our in-depth qualitative conversations extend this previous research by showing that adherence to PHM alleviates some of the anxiety young adults experience regarding the uncertainty in the COVID-19 pandemic.

However, the novelty of the measures wears as young adults become accustomed to the risk. These findings support the use of PHMs, such as circuit breakers, only when necessary; we propose that pandemic-related PHMs should be seen akin to valuable ‘non-renewable resources’ that should be ‘protected’ to ensure the adherence of young adults when necessary.

Additionally, when participants perceived the disruption caused by adherence as manageable and felt confident in their ability to adhere, they were likely to follow PHM. This finding supports other studies which show that self-efficacy and the similar concept of perceived-behavioural control drive adherence of the general population in the COVID-19 pandemic as well as other pandemics [45–47]. Our analysis elaborates on the cause of self-efficacy in a pandemic, demonstrating that self-efficacy in part arises from seeing an adherence behaviour, such as mask-wearing, modelled by others. Further, receiving clear direction from the government and public health officials on how to adhere to public health measures makes adherence easier. For example, seeing others adhere prompts young adults to adhere. This supports non-pandemic experimental work [48] as well as pandemic work [49], that shows conforming to peers’ level of adherence is a key driver of an individual’s obedience to the authority requests.

In contrast, the use of vague guidelines in explaining new rules, hinders adherence. When guidelines are vague, young adults employ personal judgement, deciding which friends are safe to interact with. This highlights the value in using transparent, clear, and concise messaging for public health guidelines, and complementing this with the use of examples of other jurisdictions with similar measures. Such approach has also been suggested by other studies [50, 51]; and we propose, given our findings, using such approach will increase the perceived self-efficacy and thus, adherence to PHMs.

We also demonstrate that perceiving non-adherence as morally culpable and perceiving a duty towards one’s community motivated adherence among low-risk young adults. Civil duty is a known motivator of adherence to protective health behaviour [39]. Our study expands on these concepts by demonstrating that feelings of civil duty are combined, for many, with significant anxiety about and a culpability to spreading COVID-19 to others; this is exemplified through the participants’ description of spreading COVID-19 as similar to texting while driving. While stigma indirectly promoted adherence, it is also important to note that stigma can lead to other social consequences such as tension between vaccinated and unvaccinated individuals [52].

The feelings of moral culpability were contextualized by the low number of COVID-19 cases in PEI, as young adults describe not wanting to seed an outbreak. We suggest that it is easier for young adults to make the necessary sacrifices to adhere when it does not appear to be a lost cause (i.e., their adherence will maintain a low-risk environment). This application adds to other adherence studies that focus on the general population (i.e., including those at high risk of infection), which proposed perception of risk [3] and personality traits [15, 53] as prominent reasons for adherence. Therefore, our findings highlight the value in utilizing public health messaging that employs a moral imperative to adhere, complemented with positive messaging when cases are low, to foster pride in the outcome of adherence.

Perceiving a collective duty to adhere was also a motivator of adherence related but distinct to moral culpability. Specifically, these young adults described the need for collectivity as stemming in part from an underlying fragile healthcare system. This finding supports a time-dynamic analysis of Europe [54] that revealed that regions with high levels of trust in the government but low confidence in the healthcare system (i.e., low expectation that sufficient and appropriate treatment will be provided when needed) dramatically reduced mobility in response to the pandemic restrictions.

There are limitations to the study that must be considered. First, socio-psychological factors such as attitudes are not always reflective of behaviour [55]. While this study focused on eliciting the attitudes and the self-reported behaviours, the relationship between the two concepts is complex and non-linear. Future research could directly observe how young adults do and do not adhere. Second, participants were interviewed at a single point in time (April-May 2021) and thus, their views do not reflect the later stages of the pandemic. Additionally, other studies could explore how adherence plays out in other populations who differ from our population in dimensions such as race, gender, sexuality, and indigeneity, as studies have shown the lived pandemic experience to differ across these populations [10, 56–58]. Further, while our findings provide details on experiences and perspectives, they are not statistically generalizable. However, we used Nowell’s criteria of trustworthiness (credibility, transferability, dependability, confirmability) [59] to ensure we provided meaningful and useful results. Future research should focus on replicating these finding in other communities, in order to strengthen the understanding on how cultural elements shape adherence in other low-risk settings.

In conclusion, this study provides qualitative insights into why low-risk young adults, an important segment of the population whose adherence is critical to the success of PHMs, follow public health guidelines. These themes support reasons for adherence reported by other studies [7, 46, 53, 54] that sampled the general population. However, our results extend this research by providing insights on the adherence of young adults in a low-risk context, with such cases of adherence being crucial for the understanding, success, and broader application of PHMs. We expand on the available literature by emphasising that adherence is also connected to moral culpability and trust in leadership, which is developed by clear and consistent communication. Therefore, we highlight the efficacy of clear PHMs that employ a moral imperative to adhere and are delivered by trusted public health officials; this delivery should be complemented with sentiments that foster pride and community, in order to encourage adherence among young adults at low risk of COVID-19 infection or adverse outcomes of infection.

Providing such insight is of high value as COVID-19 moves from a pandemic state to an endemic state, as there will continue to be a need for motivating adherence among vaccinated individuals who perceive themselves to be low risk in future outbreaks of COVID-19 variants. Conducting research along these objectives is also important for informing future pandemics, which are expected to become both more frequent and more severe owing to the impacts of globalization and climate change [60, 61].

## Supporting information

S1 Appendix Description of public health measures in Prince Edward Island. (DOCX)

S2 Appendix Pre-interview questionnaire and semi-structured interview guide. (DOCX)

S3 Appendix Codebook. (DOCX)

## References

[1] WHO Director-General’s opening remarks at the media briefing on COVID-19 [Internet]. World Health Organization; 2020 [cited 2020 Oct 1]. Available from: https://www.who.int/dg/speeches/d etail/who-director-general-s-opening-remarks-at-the-media-briefing-on-covid- 19–11-march-2020

[2] Webster RK, Brooks SK, Smith LE, Woodland L, Wessely S, Rubin GJ. How to improve adherence with quarantine: rapid review of the evidence. Public Health. 2020 May;182:163–9. doi: 10.1016/j.puhe.2020.03.007 32334182 PMC7194967

[3] Leppin A, Aro AR. Risk Perceptions Related to SARS and Avian Influenza: Theoretical Foundations of Current Empirical Research. Int J Behav Med. 2009 Mar;16(1):7–29. doi: 10.1007/s12529-008-9002-8 19214752 PMC7090865

[4] Rothstein MA, Coughlin CN. Ensuring Compliance With Quarantine by Undocumented Immigrants and Other Vulnerable Groups: Public Health Versus Politics. Am J Public Health. 2019 Sep;109(9):1179–83. doi: 10.2105/AJPH.2019.305201 31318598 PMC6687239

[5] Anderson RM, Heesterbeek H, Klinkenberg D, Hollingsworth TD. How will country-based mitigation measures influence the course of the COVID-19 epidemic? The Lancet. 2020 Mar;395(10228):931–4. doi: 10.1016/S0140-6736(20)30567-5 32164834 PMC7158572

[6] Barber SJ, Kim H. COVID-19 Worries and Behavior Changes in Older and Younger Men and Women. Isaacowitz D, editor. J Gerontol Ser B. 2021 Jan 18;76(2):e17–23. doi: 10.1093/geronb/gbaa068 32427341 PMC7313781

[7] Brouard S, Vasilopoulos P, Becher M. Sociodemographic and Psychological Correlates of Compliance with the COVID-19 Public Health Measures in France. Can J Polit Sci. 2020 Jun;53(2):253–8.

[8] Pfattheicher S, Nockur L, Böhm R, Sassenrath C, Petersen MB. The Emotional Path to Action: Empathy Promotes Physical Distancing and Wearing of Face Masks During the COVID-19 Pandemic. Psychol Sci. 2020 Nov;31(11):1363–73. doi: 10.1177/0956797620964422 32993455

[9] Moore RC, Lee AY, Hancock JT, Halley MC, Linos E. Age-Related Differences in Experiences With Social Distancing at the Onset of the COVID-19 Pandemic: A Computational and Content Analytic Investigation of Natural Language From a Social Media Survey. JMIR Hum Factors. 2021 Jun 9;8(2):e26043. doi: 10.2196/26043 33914689 PMC8191726

[10] Moyser M. Gender differences in mental health during the COVID-19 pandemic [Internet]. Statistics Canada; 2020 Jul. Available from: https://www150.statcan.gc.ca/n1/en/pub/45-28-0001/2020001/article/00047-eng.pdf?st=3DYOV4YZ

[11] de Noronha N, Moniz M, Gama A, Laires PA, Goes AR, Pedro AR, et al. Non-adherence to COVID-19 lockdown: who are they? A cross-sectional study in Portugal. Public Health. 2022 Oct;211:5–13. doi: 10.1016/j.puhe.2022.07.001 35988506 PMC9271418

[12] Nivette A, Ribeaud D, Murray A, Steinhoff A, Bechtiger L, Hepp U, et al. Non-compliance with COVID-19-related public health measures among young adults in Switzerland: Insights from a longitudinal cohort study. Soc Sci Med. 2021 Jan;268:113370. doi: 10.1016/j.socscimed.2020.113370 32980677 PMC7493799

[13] Chen C, Frey CB, Presidente G. Culture and contagion: Individualism and compliance with COVID-19 policy. J Econ Behav Organ. 2021 Oct;190:191–200. doi: 10.1016/j.jebo.2021.07.026 34566218 PMC8452375

[14] Wismans A, Letina S, Wennberg K, Thurik R, Baptista R, Burke A, et al. The role of impulsivity and delay discounting in student compliance with COVID-19 protective measures. Personal Individ Differ. 2021 Sep;179:110925. doi: 10.1016/j.paid.2021.110925 34866724 PMC8631574

[15] Miguel FK, Machado GM, Pianowski G, Carvalho L de F. Compliance with containment measures to the COVID-19 pandemic over time: Do antisocial traits matter? Personal Individ Differ. 2021 Jan;168:110346. 10.1016/j.paid.2020.110346 PMC7441860 32863507

[16] Statistics Canada. COVID-19 daily epidemiology update [Internet]. Statistics Canada. 2021 [cited 2021 Jan 15]. Available from: https://health-infobase.canada.ca/covid-19/epidemiological-summary-covid-19-cases.html

[17] Lockwood B, Taylor J. School Nutrition Policy Adherence and Weight Status in Elementary School Children in Prince Edward Island [Internet] [Thesis Dissertation]. University of Prince Edward Island; Available from: https://core.ac.uk/download/pdf/70396429.pdf

[18] Park CL, Russell BS, Fendrich M, Finkelstein-Fox L, Hutchison M, Becker J. Americans’ COVID-19 Stress, Coping, and Adherence to CDC Guidelines. J Gen Intern Med. 2020 Aug;35(8):2296–303. doi: 10.1007/s11606-020-05898-9 32472486 PMC7259430

[19] Wrzus C, Hänel M, Wagner J, Neyer FJ. Social network changes and life events across the life span: A meta-analysis. Psychol Bull. 2013;139(1):53–80. doi: 10.1037/a0028601 22642230

[20] Cohen AK, Hoyt LT, Dull B. A Descriptive Study of COVID-19–Related Experiences and Perspectives of a National Sample of College Students in Spring 2020. J Adolesc Health. 2020 Sep;67(3):369–75. doi: 10.1016/j.jadohealth.2020.06.009 32593564 PMC7313499

[21] Kuiper ME, de Bruijn AL, Reinders Folmer C, Olthuis E, Brownlee M, Kooistra EB, et al. The Intelligent Lockdown: Compliance with COVID-19 Mitigation Measures in the Netherlands. SSRN Electron J [Internet]. 2020 [cited 2022 Jan 2]; Available from: https://www.ssrn.com/abstract=3598215

[22] Zajenkowski M, Jonason PK, Leniarska M, Kozakiewicz Z. Who complies with the restrictions to reduce the spread of COVID-19?: Personality and perceptions of the COVID-19 situation. Personal Individ Differ. 2020 Nov;166:110199. doi: 10.1016/j.paid.2020.110199 32565591 PMC7296320

[23] Nebehay S. WHO message to youth on coronavirus: “You are not invincible.” Reuters [Internet]. 2020 Mar 20 [cited 2020 Jul 20]; Available from: https://www.reuters.com/article/us-health-coronavirus-who/who-message-to-youth-on-coronavirus-you-are-not-invincible-idUSKBN21733O

[24] Boehmer TK, DeVies J, Caruso E, van Santen KL, Tang S, Black CL, et al. Changing Age Distribution of the COVID-19 Pandemic—United States, May–August 2020. MMWR Morb Mortal Wkly Rep. 2020 Oct 2;69(39):1404–9. doi: 10.15585/mmwr.mm6939e1 33001872 PMC7537561

[25] Sandelowski M. Using Qualitative Research. Qual Health Res. 2004 Dec;14(10):1366–86. doi: 10.1177/1049732304269672 15538005

[26] Gill P, Stewart K, Treasure E, Chadwick B. Methods of data collection in qualitative research: interviews and focus groups. Br Dent J. 2008 Mar;204(6):291–5. doi: 10.1038/bdj.2008.192 18356873

[27] Mackay C. Call for capital region 20-somethings to get tested leads to confusion, lineups. Canadian Broadcast Company [Internet]. 2020 Dec 6 [cited 2021 Jan 15]; Available from: https://www.cbc.ca/news/canada/prince-edward-island/pei-20-29-year-olds-in-charlottetown-should-get-tested-1.5830706

[28] Heckathorn DD. Respondent-Driven Sampling: A New Approach to the Study of Hidden Populations. Soc Probl. 1997 May;44(2):174–99.

[29] Cai X, Cebollada J, Cortiñas M. A grounded theory approach to understanding in-game goods purchase. Jankowski J, editor. PLOS ONE. 2022 Jan 27;17(1):e0262998. doi: 10.1371/journal.pone.0262998 35085336 PMC8794092

[30] Zoom Video Communications Inc. Zoom Video Communications Inc. [Internet]. 2016. Available from: https://zoom.us

[31] Archibald MM, Ambagtsheer RC, Casey MG, Lawless M. Using Zoom Videoconferencing for Qualitative Data Collection: Perceptions and Experiences of Researchers and Participants. Int J Qual Methods. 2019 Jan 1;18:160940691987459.

[32] Oliffe JL, Kelly MT, Gonzalez Montaner G, Yu Ko WF. Zoom Interviews: Benefits and Concessions. Int J Qual Methods. 2021 Jan;20:160940692110535.

[33] Given L. Memos and Memoing. In: The SAGE Encyclopedia of Qualitative Research Methods [Internet]. 2455 Teller Road, Thousand Oaks California 91320 United States: SAGE Publications, Inc.; 2008 [cited 2022 Feb 22]. Available from: http://methods.sagepub.com/reference/sage-encyc-qualitative-research-methods/n260.xml

[34] Braun V, Clarke V. Using thematic analysis in psychology. Qual Res Psychol. 2006 Jan;3(2):77–101.

[35] Braun V, Clarke V. Thematic analysis: a practical guide to understanding and doing. 1st ed. Thousand Oaks: SAGE Publications; 2021.

[36] Michie S. Making psychological theory useful for implementing evidence based practice: a consensus approach. Qual Saf Health Care. 2005 Feb 1;14(1):26–33. doi: 10.1136/qshc.2004.011155 15692000 PMC1743963

[37] Michie S, van Stralen MM, West R. The behaviour change wheel: A new method for characterising and designing behaviour change interventions. Implement Sci. 2011 Dec;6(1):42. doi: 10.1186/1748-5908-6-42 21513547 PMC3096582

[38] Heslehurst N, Newham J, Maniatopoulos G, Fleetwood C, Robalino S, Rankin J. Implementation of pregnancy weight management and obesity guidelines: a meta-synthesis of healthcare professionals’ barriers and facilitators using the Theoretical Domains Framework: Implementing pregnancy weight guidelines. Obes Rev. 2014 Jun;15(6):462–86. 24629076 10.1111/obr.12160

[39] Burgess C, Wright AJ, Forster AS, Dodhia H, Miller J, Fuller F, et al. Influences on individuals’ decisions to take up the offer of a health check: a qualitative study. Health Expect. 2015 Dec;18(6):2437–48. doi: 10.1111/hex.12212 24889817 PMC5810718

[40] Paul JL, Leslie H, Trainer AH, Gaff C. A theory-informed systematic review of clinicians’ genetic testing practices. Eur J Hum Genet. 2018 Oct;26(10):1401–16. doi: 10.1038/s41431-018-0190-7 29891880 PMC6138746

[41] Collet JP, Sarmast H, Kissoon N, Mosavianpour M. Theoretical domains framework to assess barriers to change for planning health care quality interventions: a systematic literature review. J Multidiscip Healthc. 2016 Jul;Volume 9:303–10. doi: 10.2147/JMDH.S107796 27499628 PMC4959766

[42] Atkins L, Francis J, Islam R, O’Connor D, Patey A, Ivers N, et al. A guide to using the Theoretical Domains Framework of behaviour change to investigate implementation problems. Implement Sci. 2017 Dec;12(1):77. doi: 10.1186/s13012-017-0605-9 28637486 PMC5480145

[43] Cohen DJ, Crabtree BF. Evaluative Criteria for Qualitative Research in Health Care: Controversies and Recommendations. Ann Fam Med. 2008 Jul 1;6(4):331–9. doi: 10.1370/afm.818 18626033 PMC2478498

[44] Fletcher AJ. Applying critical realism in qualitative research: methodology meets method. Int J Soc Res Methodol. 2017 Mar 4;20(2):181–94.

[45] Roma P, Monaro M, Muzi L, Colasanti M, Ricci E, Biondi S, et al. How to Improve Compliance with Protective Health Measures during the COVID-19 Outbreak: Testing a Moderated Mediation Model and Machine Learning Algorithms. Int J Environ Res Public Health. 2020 Oct 4;17(19):7252. doi: 10.3390/ijerph17197252 33020395 PMC7579153

[46] Smith LE, Amlȏt R, Lambert H, Oliver I, Robin C, Yardley L, et al. Factors associated with adherence to self-isolation and lockdown measures in the UK: a cross-sectional survey. Public Health. 2020 Oct;187:41–52. doi: 10.1016/j.puhe.2020.07.024 32898760 PMC7474581

[47] Al-Sabbagh MQ, Al-Ani A, Mafrachi B, Siyam A, Isleem U, Massad FI, et al. Predictors of adherence with home quarantine during COVID-19 crisis: the case of health belief model. Psychol Health Med. 2022 Jan 2;27(1):215–27. doi: 10.1080/13548506.2021.1871770 33427487

[48] Andrighetto G, Grieco D. Peer effects on compliance with extortive requests. Brañas-Garza P, editor. PLOS ONE. 2020 Apr 24;15(4):e0231879. doi: 10.1371/journal.pone.0231879 32330154 PMC7182209

[49] Stapleton A, McCloskey C, McHugh L. Exploring the relationships between rule-governed behavior and adherence to guidelines aiming to reduce the spread of COVID-19. J Context Behav Sci. 2022 Jul;25:73–7. doi: 10.1016/j.jcbs.2022.06.005 35756099 PMC9212991

[50] Schneider CR, Freeman ALJ, Spiegelhalter D, van der Linden S. The effects of quality of evidence communication on perception of public health information about COVID-19: Two randomised controlled trials. Tanimoto J, editor. PLOS ONE. 2021 Nov 17;16(11):e0259048. doi: 10.1371/journal.pone.0259048 34788299 PMC8598038

[51] Benham JL, Lang R, Kovacs Burns K, MacKean G, Léveillé T, McCormack B, et al. Attitudes, current behaviours and barriers to public health measures that reduce COVID-19 transmission: A qualitative study to inform public health messaging. Capraro V, editor. PLOS ONE. 2021 Feb 19;16(2):e0246941. doi: 10.1371/journal.pone.0246941 33606782 PMC7895406

[52] Korn L, Böhm R, Meier NW, Betsch C. Vaccination as a social contract. Proc Natl Acad Sci. 2020 Jun 30;117(26):14890–9. doi: 10.1073/pnas.1919666117 32541033 PMC7334515

[53] Lachowicz-Tabaczek K, Kozłowska MA. Being others-oriented during the pandemic: Individual differences in the sense of responsibility for collective health as a robust predictor of compliance with the COVID-19 containing measures. Personal Individ Differ. 2021 Dec;183:111138. doi: 10.1016/j.paid.2021.111138 34511682 PMC8416600

[54] Chan HF, Brumpton M, Macintyre A, Arapoc J, Savage DA, Skali A, et al. How confidence in health care systems affects mobility and compliance during the COVID-19 pandemic. Capraro V, editor. PLOS ONE. 2020 Oct 15;15(10):e0240644. doi: 10.1371/journal.pone.0240644 33057450 PMC7561184

[55] LaPiere RT. Attitudes vs. Actions. Soc Forces. 1934 Dec 1;13(2):230–7.

[56] Ibarra-Nava I, Flores-Rodriguez KG, Ruiz-Herrera V, Ochoa-Bayona HC, Salinas-Zertuche A, Padilla-Orozco M, et al. Ethnic disparities in COVID-19 mortality in Mexico: A cross-sectional study based on national data. Hodges MH, editor. PLOS ONE. 2021 Mar 10;16(3):e0239168. doi: 10.1371/journal.pone.0239168 33690607 PMC7946310

[57] Muhajarine N, Adeyinka DA, McCutcheon J, Green KL, Fahlman M, Kallio N. COVID-19 vaccine hesitancy and refusal and associated factors in an adult population in Saskatchewan, Canada: Evidence from predictive modelling. Gesser-Edelsburg A, editor. PLOS ONE. 2021 Nov 12;16(11):e0259513. doi: 10.1371/journal.pone.0259513 34767603 PMC8589208

[58] Brotto LA, Chankasingh K, Baaske A, Albert A, Booth A, Kaida A, et al. The influence of sex, gender, age, and ethnicity on psychosocial factors and substance use throughout phases of the COVID-19 pandemic. Page K, editor. PLOS ONE. 2021 Nov 22;16(11):e0259676. doi: 10.1371/journal.pone.0259676 34807908 PMC8608308

[59] Nowell LS, Norris JM, White DE, Moules NJ. Thematic Analysis: Striving to Meet the Trustworthiness Criteria. Int J Qual Methods. 2017 Dec;16(1):160940691773384.

[60] Brooks DR, Boeger WA. Climate change and emerging infectious diseases: Evolutionary complexity in action. Curr Opin Syst Biol. 2019 Feb;13:75–81.

[61] Epstein PR. Climate Change and Public Health: Emerging Infectious Diseases. In: Encyclopedia of Energy [Internet]. Elsevier; 2004 [cited 2022 Jan 2]. p. 381–92. Available from: https://linkinghub.elsevier.com/retrieve/pii/B012176480X004320

