# Supplementary material for: Adherence of those at low risk of disease to public health measures during the COVID-19 pandemic: A qualitative study
Source: PLoS One. 2022 Oct 25;17(10):e0276746. doi: 10.1371/journal.pone.0276746 (PMC9595514; doi:10.1371/journal.pone.0276746)
Supplement: S1 Appendix — (DOCX) [file pone.0276746.s001.docx]

**S1 Appendix**

**Description and Timeline of Selected Public Health Measures**

**in Prince Edward Island**

Through this supplemental, we seek to provide necessary contextual information on Prince Edward Island and the measures they used to respond to the COVID-19 pandemic. Much like a reflection on positionality, the provision of further information on the study geography and sociopolitical events during our research is critical for the readers of our paper to contextualize its finding.

Prince Edward Island (PEI) is a Canadian Island province located on the east coast of Canada. It is Canada’s smallest province geographically. The population of PEI is roughly 160,000 people at the time this research was conducted. As it is an island, there are only three ways to enter the province: the Confederation bridge, the ferry (both the Wood Island ferry, the Magdalen Island ferry), and the airport. Colloquially, the province is known for its seafood, “friendliness”, and beaches. While the province is a tourist destination, it has had difficulty in recruiting healthcare professionals to PEI. Often spots remain open for many years before being filled and emergency rooms across the province do not always operate at full capacity (CBC News, 2021). Such information sets the stage for the events that took place during the COVID-19 pandemic.

In March 2020, the Premier of PEI, Dennis King, declared the province to be in a state of emergency (Figure 1). This action paralleled the action of other Canadian provinces. King also announced that the pandemic response in PEI would be directed solely by Dr. Health Morrison, the chief public health officer of the province. Throughout the pandemic, the King government, under the direction of Dr. Morrison would go on to announce several public health measures (PHMs) that aimed to (1) prevent COVID-19 from entering the island and (2) prevent the spread of COVID-19 on PEI.

The qualitative research conducted on young adults in PEI focuses on five PHMs used in PEI during the COVID-19 pandemic, namely 14-day travel quarantine, social distancing, masks, circuit breakers, and vaccinations. The choice of these measures reflects their prominence in PEI’s pandemic response.

***14-Day Travel Quarantine***

Early on in the pandemic (March/April 2020), PEI mandated a 14-travel quarantine. That meant that everyone entering PEI (both residents and non-residents) were required to complete a 14-day quarantine/isolation period. The implementation of this measure was new as it had not been done in PEI. When this measure was initially implemented, there was less guidance on exactly what a quarantine meant. However, the province quickly developed tools to ensure that quarantine became a well-adhered and legally enforced measure. Specifically, when travelers arrived at each border entry, they were advised of the need to isolate and requested to provide a phone number. Travellers were then called every day for the full 14-day isolation; these calls included symptoms checking and the provision of social or health-related support when needed.

As COVID-19 testing became available, the province began testing travellers in the middle of their self-isolation. And later when antigen testing became available, travellers were requested to test on entry to the province. Since there are only three ways to enter the province (bridge, ferry, airport), PEI was able to enforce testing of all travellers, once testing became available. This approach allowed the province to effectively eliminate community spread.

***Social Distancing***

In addition to travel-related quarantine, social distancing was also implemented by government officials in order to reduce the spread of COVID-19. Social distancing included both physical distancing (i.e., keeping a 2-meter apart) and gathering limitations both in public settings as well as private gatherings. These measures changed throughout the pandemic and ranged from bans on gatherings with those outside of your household to allow gatherings of up to 50 people indoors. The gathering PHMswere particularly stringent during periods of high COVID-19 case counts and were most relaxed during the summer months when case counts were low throughout Canada.

***Masks***

In Canada, mask mandates were delivered at the provincial level. Throughout the majority of 2020, PEI deemed masks as a “recommended” measure, and masks were to be used discretionarily. However, masks became mandatory in grocery stores in the early summer 2020. In November 2020, PEI officially mandated masks in all in all indoor public spaces, making PEI the last Canadian province to do so. Masks remained mandatory up to and including the time of data collection (April-May 2021) and manuscript preparation

***Circuit Breaker***

While the aforementioned measures focused on preventing the occurrence of community spread, “circuit breakers” were designed to control and eliminate community spread. Circuit breakers referred to a two-week lockdown where only essential activities and gatherings were allowed to take place. The term circuit breaker was a new term to residents of PEI; all participants describe being unaware of such measure prior to its first official use. The first circuit breaker occurred in December 2020 in response to the first detection of community spread in PEI. Until that point, all previous COVID-19 cases on PEI could be traced to travel.

At the beginning of this circuit breaker, the PEI government released a list of public exposure locations for COVID-19. This allowed individuals to self-identify if they were in proximity to a COVID-19 case. While this measure was implemented in the first circuit breaker, the provincial government continued to use it throughout the remainder of the pandemic. Also, during the circuit breaker, the PEI government requested that all young adults get a COVID-19 PCR (polymerase chain reaction) test as the majority of the community spread cases had been in their 20s. Such an overwhelming number of young adults showed up to be tested (line-ups were 7 hours long) that the province had to alter their request the following day to “only those in their 20s living in the Charlottetown area” and then further the following day to “… and if symptomatic”.

The first circuit breaker lasted from December 7^th^-21^st^ 2020, just shy of two weeks in length. The original duration was proposed to be two-weeks; however, it became clear that there was no evidence of community spread and thus, measures were lifted early.

The second circuit breaker was put in place in February 2021, after the province reported 11 new COVID-19 cases over the course of one weekend. This circuit breaker was much shorter, lasting only a few days as it became clear that the outbreak was well-controlled, with cases only being limited to the Summerside community. During this circuit breaker, only individuals exposed in the Summerside community or showing symptoms were asked to get tested.

***Vaccinations***

On December 16, 2020, the first doses of Pfizer-BioNTech were administered to residents, mostly to healthcare workers, in PEI. The vaccination rollout strategy PEI had two streams: (1) targeting healthcare workers (primarily with the Pfizer-BioNTech and Moderna vaccines) and (2) front-line workers between the ages of 16 and 39 (primarily AstraZeneca). The later stream began in March 2021 and was subsequently altered following the discovery of post-AstraZeneca complications. By May 5^th^ 2021 (the time the data for this study was collected), over 30% of Islanders over the age of 12 had received their first dose of the vaccine. Therefore, during the semi-structured interviews, either the desire to be vaccinated or whether the participant was vaccinated was elicited based on their eligibility according to PEI’s vaccine rollout plan. Most were not eligible at the time of data collection. However, by November 2021, the PEI government reported that over 90% of all Islanders had been fully vaccinated against COVID-19.


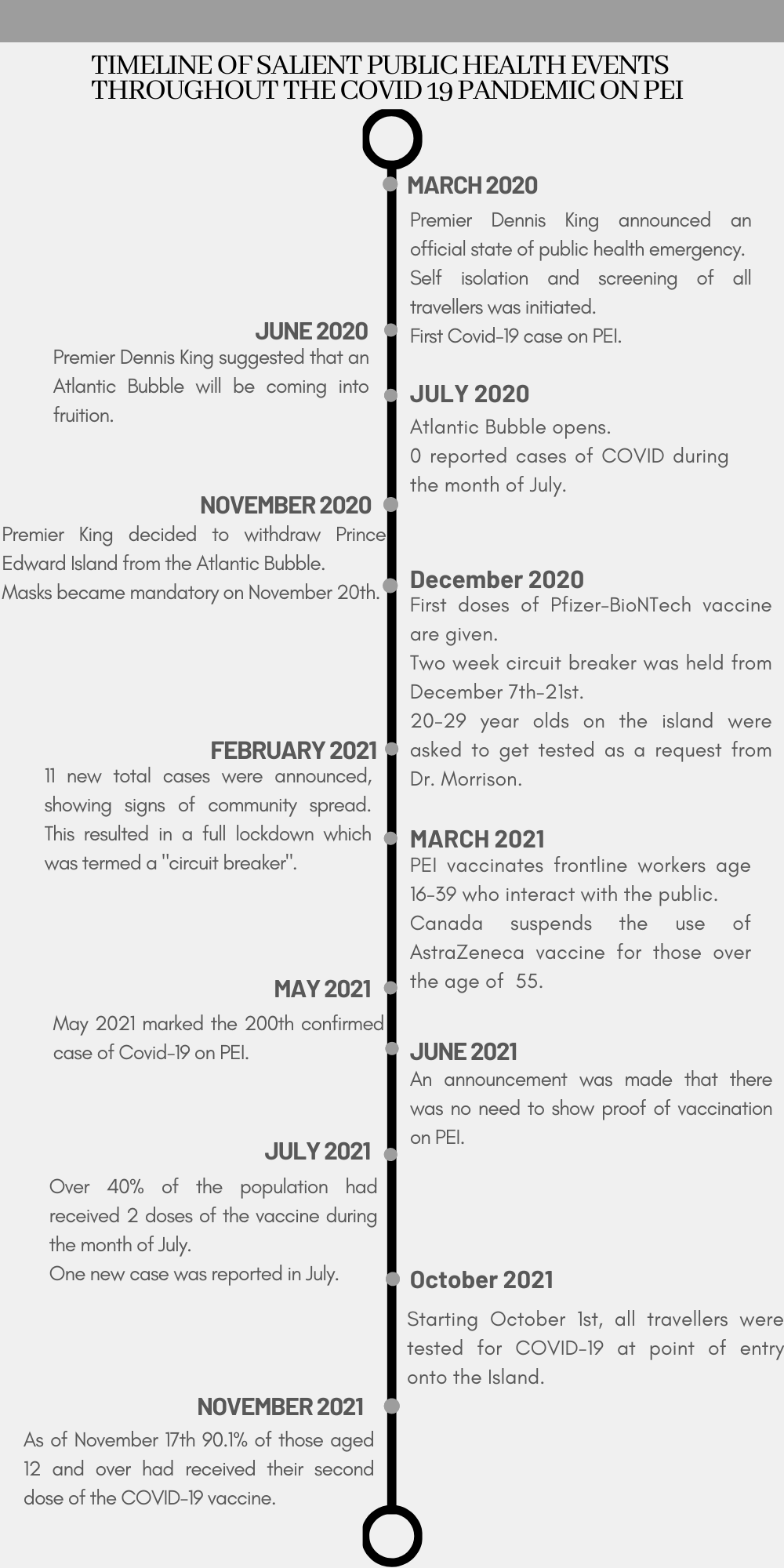


**Supplemental Figure 1**: Timeline of salient public health events throughout the COVID-19 pandemic on Prince Edward Island (PEI).

*This figure outlines a timeline of all the relevant public health events that occurred in PEI during the first part of the COVID-19 pandemic (March 2020 and November 2021). Important information regarding public health measures and case counts were included in this schematic.*

**References**

# CBC News (2021). “'No quick fix' to staffing shortages, CEO of Health PEI says.” https://www.cbc.ca/news/canada/prince-edward-island/pei-health-changes-shortages-1.6236247
