# Supplementary material for: Adherence of those at low risk of disease to public health measures during the COVID-19 pandemic: A qualitative study
Source: PLoS One. 2022 Oct 25;17(10):e0276746. doi: 10.1371/journal.pone.0276746 (PMC9595514; doi:10.1371/journal.pone.0276746)
Supplement: S2 Appendix — (DOCX) [file pone.0276746.s002.docx]

**S2 Appendix**

**Questionnaire for Interviewees**

Experiences of young adults living in PEI during the pandemic

*Please complete the following questions to the best of your ability. All information recorded will remain confidential.*

**Demographic Questions**

| 1) What is your age? ___________ | 2) What is your gender? ___________ |
| --- | --- |

**Education and Employment Questions**

| 3) What is your highest level of education?  Less than high school High school  College Diploma / Undergraduate degree  Masters or Post-graduate degree  4) Are you currently a College / University Student?  Yes No | 5a) Are you currently employed?  Full-Time Part-Time Not Employed  5b) If so, what is your occupation?  _______________________________  5c) Does your occupation involve direct contact with animals?  Yes No |
| --- | --- |

**Residence and Family Questions**

| 6a) What is your postal code?  _______________________________    6b) What type of dwelling do you live in?  Condominium/Apartment  Duplex/Townhouse  Detached House  7) What is your marital status?  Married  Cohabitating (common law)  Separated/Divorced  Widowed  Never Married | 8a) How many people do you live with? ___________  8b) How many of the people you live with are older than 60 years? __________  8d) Do you live with people who identify as immuno-compromised?  Yes No  9a) How many children do you have? _________  9b) Do your children live in your household?  Yes No  9c) What are the ages of your children? |
| --- | --- |

**Information Questions**

| 10) What is your primary source of news information?  Facebook  Twitter  Other Social Media Sources  Island Newspapers or News Channels  Off-Island News Platforms  Family/Friends  Other _____________ | 11) What is your primary source of COVID-19 information?  Facebook  Twitter  Other Social Media Sources  Island Newspapers or News Channels  Off-Island News Platforms  Family/Friends  Other _____________ |
| --- | --- |
|  |  |

**COVID-19 Related Questions**

*Please check either the box labeled true or false based on statement provided.*

| **Statement** | **True** | **False** |
| --- | --- | --- |
| 1. COVID-19 spreads through respiratory droplets, which occur when infected people cough and sneeze. |  |  |
| 1. COVID-19 can be contracted by touching a surface or object on which the virus is attached. |  |  |
| 1. Close contact with or eating wild animals causes COVID-19. |  |  |
| 1. The main clinical symptoms of COVID-19 are fever, fatigue, dry cough, and loss of sense of small, and shortness of breath. |  |  |
| 1. Antibiotics are an effective treatment for COVID-19. |  |  |
| 1. Older adults and those with serious chronic illnesses, such as heart or lung disease and diabetes, are at increased risk of developing more serious complications from COVID-19. |  |  |
| 1. Pets cannot get COVID-19. |  |  |

**Semi-structured Interview Question Guide**

Experiences of young adults living in PEI during the pandemic

**PART 1) Consent form and questionnaire**

*We are here today to talk about COVID-19, and we are especially interested in the experience of young people on PEI. A main part of this interview will be that I ask you questions about your experience with the COVID-19 pandemic and I will audio-record our conversation. However, I want to highlight that your identity will remain confidential and you can terminate the interview at any time without repercussions. Further, you do not have to answer any of my questions if you do not feel comfortable doing so. To be able to participate in this study, I need your consent. If you not have already returned a signed copy of the consent form to me, we will now go through it.*

*Read through consent form*

*Do you have any questions about this? If not, do you agree to participate in this study?*

*We are first going to get you to fill out this short questionnaire so that we understand about you and also how familiar you are with COVID-19. Again, your* *identity will remain confidential.*

*Fill out questionnaire*

**PART 2) Perceptions of and Overall Adherence to the COVID-19 Regulations**

*Now, I am going to ask you some questions about your experience with the COVID-19 pandemic. Please answer the questions as honestly as possible. Again, your identity will remain confidential.*

- *Can you describe to me how you experienced the COVID-19 pandemic in your day-to day life since it began early in 2020?*
  - *What has changed in your daily routine?*
  - *How did people that are around your age in PEI experience the COVID-19 pandemic?*
- *How do you feel about the risks of COVID-19?*
  - *To you personally?*
  - *To other people?*
  - *To your pets?*
  - *How did these feelings change throughout the pandemic?*
    - *i.e., did you feel the risks were greater or less at the beginning?*
    - *i.e, now that people are starting to be vaccinated, do you feel the risks are greater or less than at the beginning?*
- *Can you tell me some of the regulations that were put in place in PEI to reduce the spread of Covid-19?*
  - *What is your opinion of these regulations?*
    - *At the time these regulations were put in place, do you think they were necessary? Why (not)?*
    - *How about now, do you think the regulations were necessary? Why (not)?*
    - *Do you think some regulations were more effective than others? Which ones? Why?*
  - *As people are being vaccinated, do you think they are still necessary?*
- *Did you follow the regulations? Why (not)?*
  - *Can you tell me a situation where it was difficult for you to follow the regulations?*
  - *Without mentioning names, do you know to what extend other people your age followed the regulations?*

*What might have been reasons they did not follow the regulations?*

- - - *How do you feel about that?*

*What might have been reasons they followed the regulations?*

- - - *How do you feel about that?*
  - *If you were to rate your compliance on a scale of 1 to 10 (1 being you did not follow any of the rule if they required change to your routine and 10 being you followed every rule perfectly), how would you rate your compliance?*
- *Did you ever get a COVID-19 test?*
  - *Why or why not?*
  - *Did you follow up on your test results?*

**PART 3) Specifying Measures of Compliance in the Circuit Breaker**

*Now, we will move onto the third part where we would like to ask you a few questions, specifically about the circuit breakers. In response to non-travel linked cases in December 2020 and again in February 2021, Public Health PEI announced circuit breakers. The one in December lasted two-week circuit breaker while the one in February/March was three days.*

- *Do you remember what measures were put in place as part of the circuit breaker? If not, I can give you a more detailed overview.*
  - *Answer: Yes 🡪 Continue with the interview as follows*
  - *Answer: No 🡪 Read ‘Additional Information’ at the bottom*
    - *Do you remember the circuit breaker now that you have received the additional information?*
      - *Answer: No 🡪 Terminate interview (PART 4)*
      - *Answer: Yes 🡪 Continue with the interview as follows:*
- *What was your opinion of each circuit breaker at that time they were announced?*
  - *Did you believe at the time that the circuit breakers were necessary?*
  - *Why or why not?*
- *In both circuit breakers exposure locations were publicly known (for example, ‘potential exposure at the Gahan House from 7:30-9pm’). How did this make you feel?*
  - *Did that affect your likelihood of going to these locations?*
    - *Has this gone back to normal since?*
- *Many 20-year-olds were tested as a precautionary measure at the beginning of both circuit breakers. Did you get a COVID-19 test? Why or Why not?*
  - *Answer: No 🡪 Proceed to the next set of questions*
  - *Answer: Yes 🡪 Continue:*
    - *How was that experience?*
    - *What motivated you to get a COVID-19 test?*
    - *At the time did you feel it was necessary?*
- *In December, how did this announcement change who you would have interacted with in the following two weeks?*
  - *Were these impacts the same in February?*
- *In December, were other people your age following the rules?*
  - *How did that affect you?*
  - *Do you think this changed in February?*
    - *Did their following the rules affect you more or less in February? Why?*
- *Did you feel that there would have been consequences if you did not follow the rules in either of the circuit breakers?*
  - *What types of consequences?*
  - *Do you think others felt this way?*

**PART 4) Vaccine Adherence**

*Now, we will move onto the fourth part where we would like to ask you a few questions, specifically about the COVID-19 vaccines.*

- *The Pfizer-BioNTech and Moderna COVID-19 vaccine were both approved in December 2020; the AstraZeneca vaccine was later approved in February 2021. Do you remember hearing about these approvals of each vaccine?*
  - *What was your initial reaction?*
    - *Did you immediate friends and family have the same reaction?*
  - *Has your initial reaction changed? Why or why not?*
- *Did you have any questions about the vaccines when they were approved?*
  - *If so, what types of questions?*
  - *Where did you look to find answers to these questions?*
  - *Did you find sufficient answers to these questions? Are their outstanding questions?*
- *Have you received a vaccine? When it is your turn do you plan on receiving one?*
  - *Why or why not?*
  - *Does your decision to get a vaccine depend on the type of vaccine being used?*
  - *Do those who you live with feel the same way?*
- *Will receiving the vaccine alter how you follow the guidelines that will be in place at the time?*
  - *Why or why not?*
  - *Can you give me an example to highlight what you mean?*
  - *Will it affect who you will interact with? (i.e., greater time with friends or more interactions with neighbours)*
- *PEI decided to vaccinate first those above the age of 80 (both doses) and then single doses to the rest of the population in order to get as many vaccinated as possible by the summer. This part was similar to the rest of the country. However, PEI also specifically targeted those 18-29 years working in the food industry. What do you think of all of these decisions?*
  - *Do you think there is a better approach?*

**PART 5) Closing**

*That concludes the more formal part of the interview.*

- *Is the anything else you want to add to the information you have provided before we terminate the interview?*

*Thank you so much for taking the time to meet with me today. Your insights are very much appreciated. If any questions about this study come up, please feel free to get in touch. We will send you a list of counselling services immediately after this interview in case you want to reach out to any of them. Additionally, we will provide you with the transcript of your interview as soon as possible and a report of the results if that is what you indicated on your consent form.*

**Additional Information about the circuit breaker that can be shared with participants**

On Monday December 7^th^ 2020, Dr. Heather Morrison (the Chief Health officer of PEI), announced that new health protocols would affect the following day. These included:

- Banning in-restaurant dining, closing of all fitness facilities and libraries, limited organized gatherings to no more than 10 people, and four high schools moved to online learning.
- Retail stores must also limit capacity to 50 per cent.
- In the announcement, Dr. Morrison included a plea for all Islanders to avoid social gatherings and remain at home. Dr. Morrison also asked all Islanders between the ages of 20 and 29 to get tested for COVID-19, even if they have no symptoms.

In February, similar measures were taken in response to 6 non-travel linked cases in Islanders in their 20s. The restrictions included:

- Schools and most non-essential businesses are closed (including restaurants and gyms)
- The new rules also require Islanders to practise physical distancing with anyone outside their immediate households. Exceptions are being made for people who live alone or require essential support.
- The Chief Public Health Office has asked all people aged 14-29 in the Summerside area to get tested this weekend even if they are not experiencing symptoms.
