# Supplementary material for: Adherence of those at low risk of disease to public health measures during the COVID-19 pandemic: A qualitative study
Source: PLoS One. 2022 Oct 25;17(10):e0276746. doi: 10.1371/journal.pone.0276746 (PMC9595514; doi:10.1371/journal.pone.0276746)
Supplement: S3 Appendix — (DOCX) [file pone.0276746.s003.docx]

# **S3 Appendix**

# **Codebook: Parent Code and Child Codes for the Eight Themes affecting young adults’ adherence to Covid-19 PHM**

| **Theme** | **Parent Codes**  Parent Code Definitions | **Child Code**  Child Code Definition |
| --- | --- | --- |
| **Clear, purpose driven adherence rationale** | **Awareness of the changing situation**  Participants sought out information and updates on the public health rules and recommendations. | **Frequenter of the news stations**  Being up-to-date and knowing all the facts was critically important for participants |
|  |  | **Critical consumption of knowledge**  Participants felt that knowledge had to be consumed critically and often sought out the principal source of the information to verify if it was true |
|  | **Utility of public health measures**  Participants felt that the public health measures would do what they were intended to do (and that this would help keep case counts low/get them back to a pre-pandemic state). | **Necessary measure because of recognized risk**  Participants felt that guidelines (and by extension) the general public's adherence was necessary to prevent a negative outcome |
|  |  | **Ability to control risk with compliance**  Participants felt that they were capable of attaining personal safety through compliance with the guidelines. However, with vaccinations, participants expressed awareness of the interventions limitations and this did not affect their likelihood of getting or desiring to get vaccinated. |
|  |  | **Measures assist with flattening the curve**  Participants believed that the public health measure (if complied to) was able to reduce the spread of COVID-19. |
|  |  | **Scientific Feat**  Participants expressed awe or respect for scientific discovery around the vaccine |
|  |  | **It will all be worth it**  Participants describe the purpose to complying to public health interventions and thus, that it will be worth it in the end.  This includes references of participants expressing that the reason for and benefit of compliance with the public health measures is returning to a pre-pandemic (or pre-circuit breaker) state, as well as references of their motivating factor for getting vaccinated being that there will be less PH rules/travel restrictions. |
| **Developing trust in the local leadership** | **Leadership has their best interest at heart**  Participants feel that the leadership has their best interests at heart | **Public Health taking the reins: Mamma Morrison**  Participants were comforted by the fact that public health called the shots for the Island's pandemic response. Participants also described holding Dr. Heather Morrison in high regard and describe her as caring & genuine (authentic) -- an esteem that they relate to being a mother |
|  |  | **Respect for Leadership's Decisions**  Participants view the decisions made by the public health officials in high regard. This includes both admiration for "strategic" and also understanding/sympathy for what could be considered a 'mistake' on the part of PH PEI. |
|  |  | **Transparency and direct communication**  Participants felt that the gov/PH displayed considerable transparency and this augmented the level of trust they felt for them.  One of the mechanisms of transparency was the delivery of the public health messaging through Dr. Heather Morrison via the briefs (both scheduled and unscheduled). Those that were unscheduled also had the effect of making participants realize that things were not good. |
|  | **Do what you're told**  Participants did as the rules and public health officials directed | **Doesn't matter if you don't agree**  Participants felt that they had to accept the direction of the government, given that it was something they could not control; However, they felt that accepting the government occurred whether they agreed with them or not. |
|  |  | **Rule follower as an identity**  Participants describe that following the rules is part of who they are and something that existed before the pandemic |
|  |  | **Groupthink**  The practice of thinking or making decisions as a group in a way that discourages creativity or individual responsibility. |
|  |  | **Not a subject matter expert**  Participants diminish their capability of making comments or critiquing the plan rolled out by the leadership because they do not have the same experience and are therefore, less capable of providing an informed opinion |
| **Adapting to novel measures** | **Difficulty complying with disrupted daily habitus**  New circumstances (including the circuit breakers) brought participants a sense of anxiety or concern | **Conscious focus on adherence when routine altered**  Participants describe how they needed to focus on the adjustments they were making to their lives to ensure that they followed the rules. This includes sentiments of forgetting a mask |
|  |  | **With practicing adhering became easier**  Participants describe that adherence to the guidelines became easier as time went on |
|  | **Confusion with vague foreign guidelines**  New guidelines (including the circuit breakers) created obstacles to adhere to guidelines as young adults lacked clear direction on what adhering looked like. | **Confusion & lack of clarity regarding new rules**  Participants were unsure about (or misinterpreted) how a guideline should be interpreted or followed |
|  |  | **Absence of a social code of conduct**  Participants describe not knowing how to navigate a social situation (what to do or say) in the pandemic. |
|  | **Constantly changing measures challenging complacency**  Participants described how they became increasingly complacent to a measure as time progressed and how (i.e., weaning novelty & increasing complacency) new measures challenged the complacency. | **COVID-19 Fatigue: Less serious the second time**  Participants felt that during the second circuit breaker, the consequences were less serious |
|  |  | **Complacency / Ebb and Flow of Compliance**  Participants described that as their perception of risk or feelings of fear lessened, they became more comfortable engaging in social activities or being in public spaces (even to the extent of non-compliance) |
|  |  | **Re-prioritizing compliance with renewed novelty**  Salient events or new information prompted participants to re-examine their priorities, the places they went, and the people they saw. |
| **Manageable Disruption** | **It wasn't that difficult**  Participants believed that they had the ability (skills, tools, support, etc.) to comply (wear a mask, self-isolate, etc.) | **All I had to do**  Participants describe the compliance as not that difficult (or even easy) and something they felt they were able to do. This includes instances where describe that the COVID-19 guidelines did not impact their day-to-day activities and thus, they were compliant by default. |
|  |  | **Comfort in seeing the behavior already done**  Participant expressed that seeing the behavior implemented and adopted in other provinces gave them a great deal of comfort |
|  |  | **We did it and that was that**  Participants describe following the guidelines simply because it was 'easy’ (i.e., it lacked the need to think for themselves or they already had the skill (or lifestyle) in place to comply). |
|  |  | **Timing made it easier**  Participants describe the timing of an event as motivating (or de-motivating) their compliance to the guidelines. |
|  | **Compliance was the normative course of action**  Participants describe how compliance became weaved into the social fabric | **Everyone is doing it**  Participants made generalized statements that implied an absence of non-likeminded people; in other words they believed in normalized behavior. |
|  |  | **Normalized Mask wearing**  Participants expressed that the public health measure became a normal aspect of their social life (and even something that may continue post-COVID). |
| **Adhering to Reduce Anxiety** | **Non-eliminable risk**  Participants felt that even living in the bubble with a lower risk, the risk of COVID-19 had not been entirely eliminated. Participants described this causing "anxiety" and increased their support of and compliance to public health measures. | **House of Cards**  Participants expressed that one small mistake could lead to massive adverse outcomes |
|  |  | **So many unknowns**  Participants referenced that there were unknown variables and that created considerable anxiety. This includes participants describing feeling anxious about the Atlantic Bubble opening. |
|  |  | **What scares me is I know it's coming**  Participants felt that the prospect of getting back to normal after COVID-19 was far away and there was need to keep diligent. |
|  | **Peace of mind thing**  Participants described that compliance provided a sense of relief from the worry and uncertainty.  This includes when participants complying to put others at ease/reduces the worry of others. | |
|  | **Didn't want to risk it**  Participants engaged in precautionary public health behaviors so as to avoid risking the negative consequences; they described their rationales as intrinsic, rather than in response to a public health measure. | |
| **Collective duty towards one's community** | **Compliance in a small town**  Participants describe the unique elements of and/or needs for compliance in a small town. | **Protecting each other**  Participants emphasized that the motivation or rationale for complying with a specific PH measure was to protect the community at large. Part of this stemmed from sentiments of having a collective culture while some of it was non-altruistic, with participants believing everyone needed to comply to maintain community openness (for themselves). |
|  |  | **Limited Resources on PEI**  Participants acknowledge the limited resources on PEI in reference to why compliance was essential |
|  | **Community pride in being relatively COVID-free**  Participants describe feeling proud of their ability to remain covid-free and the need to keep it that way. | **Envy of Canada**  Participants felt that others, living outside of PEI, viewed the Island's public health response and subsequent freedoms as a 'prize possession' |
|  |  | **Broken Window Theory**  Participants were proud of the outcome of their compliance with PEI's guidelines (i.e., no community spread) and were more inclined to continue complying because they wanted to keep it "pristine" (i.e., COVID-19 case free), as opposed to it having already gone downhill and there being spread everywhere ("Broken Windows Theory") |
| **Moral culpability** | **Consequences to non-compliance**  Participants believed that there would be repercussions of some type (social, legal, financial, etc.) if they did not follow the public health rules | **Consequence of Contracting COVID**  Participants expressed that contracting COVID would be a negative consequence of not-following the rules. |
|  |  | **Life-long guilt from spreading COVID to others**  Participants expressed that spreading COVID-19 to others would have significant, and even life-long, social/moral consequences. |
|  |  | **Legal enforcement**  Participants describe the fines and possibility of getting in trouble with the law as factors that motivated (or do not affect) their compliance |
|  |  | **2-degrees of separation: inadvertently close to a lot of people in the community**  Social media enabled participants to connect with friends and family; in other words, it facilitated the provision and reception of social support (typically emotional support) -- thus, mediating the experiences of loneliness |
|  |  | **Consequence to my professional role**  Participants felts that not complying could have negative consequences for their professional role |
|  | **Reckless** **deviance**  Participants describe that the consequence of not following the rules as being labelled reckless/irresponsible and subsequently, disappointing others. | **Labelled Irresponsible**  Participants describe that the consequence of not following the rules as being labelled a delinquent or irresponsible |
|  |  | **Disappointing others**  A secondary characteristic of being labelled the delinquent was the social shame that accompanied it as well as the knowledge that you disappointed others |
| **Using caution rather than compliance** | **Calculating a low individual risk level**  Participants describe feeling at low risk -- this was due to living in a safe and well-protected province with a low number of COVID-19 cases and the resulting absence of risk/ consequence (due to risk being sufficiently mediated not necessarily non-existent) | **Calculating the risk**  Participants described having weighted the risks of and benefits surrounding participating in an event/or going out in Public. Their outcome of the risk calculation was influenced by time and the measure discussed. (i.e., high when AZ blood clots came out) |
|  |  | **Low risk with zero COVID cases**  Participants describe a general lack of fear of getting COVID while living on the Island given that the provincial goal was to keep COVID-19 cases to zero. The lack of fear and knowledge of their low number of COVID-19 cases led them to believe that interventions weren't necessary and/or it was alright to break.  Participants were, however, still aware that a risk does exist to some people, especially those living in "hot spots". |
|  |  | **Invincibility of Youth**  Participants felts that they would be unaffected physically by the virus |
|  | **Inability to sever social connections for compliance**  Participants describe that compliance was difficult because they needed to socialize with friends/family | **Loneliness due to separation from family**  Participants describe it being challenging (or sad) to be separated from family |
|  |  | **Direct consequence of compliance is less freedom**  Participants felt that the draw back to following the rules was less freedom |
|  |  | **Need for meeting social quotidian**  Participants describe a *must* or a *need* to engaging in social environments (for mental health, for sanity, for personal enjoyment) that would interfere with their ability to follow the rules because of its personal importance |
|  | **Rule of the law vs. Spirit of the Law**  Participants describe their intention being to determine and follow the spirit of the law but not necessarily the rule of the law. | **Intergroup vs. intragroup social distancing**  Participants delineate between the need to adhere to social distancing measure when they are with friends than when they are not. |
|  |  | **Employing caution instead of consistency**  Participants delineate between the importance of keeping consistency of the number of people in their immediate social circle vs. the number of people at each gathering |
|  |  | **Changed nothing in my routine, just caution**  Participants describe that new information or new guidelines were considered and may have affected how they felt but ultimately changed nothing in how they went about their routine |
